# Integrated, Cross-Entity Information on Preventive Measures for Bowel, Breast, and Prostate Cancer: Evaluation Study of the Web Application “Prevent-Take-Up”

**DOI:** 10.2196/76393

**Published:** 2025-11-19

**Authors:** Angelika M R Kestler, Julian D Schwab, Tanja Jähnig, Michael Karl Melzer, Friedemann Zengerling, Stefan Lukac, Wolfgang Janni, Christian Bolenz, Anne Barzel, Hans A Kestler, Thomas Seufferlein

**Affiliations:** 1 Department of Internal Medicine I, Ulm University Hospital, Albert Einstein Allee 23, Ulm, 89081, Germany, +49 731 50044555; 2 Institute of Medical Systems Biology, University of Ulm, Ulm, Germany; 3 Department of General Practice and Primary Care, Ulm University Hospital, Ulm, Germany; 4 Department of Urology and Pediatric Urology, Ulm University Hospital, Ulm, Germany; 5 Department of Obstetrics and Gynecology, Ulm University Hospital, Ulm, Germany

**Keywords:** cancer prevention, website application, oncology, colorectal cancer, breast cancer, prostate cancer

## Abstract

**Background:**

About 40% of cancers are preventable through evidence-based interventions; however, uptake remains suboptimal. Knowledge and acceptance of primary and secondary preventive measures in the general population is not sufficient. We hypothesized that a web-based tool providing comprehensive, easily accessible, and individualized information on preventive strategies for multiple tumor entities could support informed decisions.

**Objective:**

This study aimed to evaluate an interactive web application offering guideline-based, risk-adapted information on preventive measures for colorectal, breast, and prostate cancer.

**Methods:**

The content of web application was developed based on German S3 guidelines. Evaluation questions consisted of the system usability scale questionnaire and queries developed by the Prevent-Take-up consortium. An initial version was tested by two focus groups comprising general practitioners (GPs), specialists and patients, revised and then made publicly available in GPs’ offices, hospitals, and pharmacies. We report on the evaluation of the revised web application. Data were collected from 2022‐2023. The web application also gathers information regarding family and individual risk factors and offers personalized recommendations (eg, to seek further information). Personal data related to specific recommendations were not stored. Participants receiving a recommendation from the web application were asked to anonymously answer questions about the information provided and the website application’s functionality using a 5-point Likert scale. As breast cancer mainly occurs in women and prostate cancer only in men, the questions regarding prevention had intrinsic sex specific items and hence all data were evaluated by sex using descriptive statistics. The main evaluation questions were (1) usability or user-friendliness of the web application for cross-entity cancer prevention and (2) motivation of users to seek further preventive advice by the information received.

**Results:**

Data from the first 101 users (62 female, 38 male, one unspecified; predominantly aged 50‐70 y) showed high score regarding user-friendliness (female 47/62, 76%; male 25/38, 65%), question comprehensibility (female 54/62, 87%; male 32/38, 83%), and the relevance of recommendations (female 47/62, 76%; male 24/38, 63%). A total of 37/62 (59%) of female and 16/38 (44%) of male participants appreciated the web application’s functionality; 29/62 (47%) of female and 14/38 (37%) of male participants reported increased knowledge about prevention and early detection of colorectal, breast, and prostate cancers. Additionally, 44/62 (71%) of female and 18/38 (47%) of male participants expressed willingness to follow-up on the web application’s recommendations and seek more information from their GPs.

**Conclusions:**

Our web application for risk-adapted prevention across multiple cancers was rated as user-friendly by participants. Having used the web application, more female participants than males were willing to seek further information on prevention and early detection measures. Overall, our study demonstrates that a web application can be a useful tool to deliver integrated and individualized prevention recommendations.

## Introduction

### Background

In 2022, cancer was the second leading cause of death in Germany after cardiovascular diseases, with 239,948 fatalities [1] and the total number of new cancer cases remains high. About 40% of all cancer cases could be prevented [2]. For some tumor entities, there are national screening and early detection programs. In case of colorectal cancer, a statutory colonoscopy screening program was established in Germany as early as 2002. This endoscopy program is complemented by the option of fecal occult blood testing. In 2019, the guaiac-based stool test was replaced by fecal immunochemical test (FIT) in the program. The whole program has reduced the cancer-specific mortality rate by around 30% over the last 20 years [3]. A similar screening program was established for breast cancer in 2004, inviting women aged 50-69 years to undergo mammography every two years. In 2023, the program was expanded to include women up to the age of 75 [4]. For prostate cancer screening, every man over the age of 45 is entitled to an annual digital examination of the prostate. However, this particular recommendation is about to be replaced. Colorectal and breast cancer screening in Germany are organized as invitation schemes that start at the designated age of eligibility. Despite these programs, there is still insufficient awareness of preventive measures and screening and a shortage of comprehensive information tools that encompass different tumor types and consider various risk-modifying factors, including smoking, obesity, alcohol consumption, and family history [5-7].

Mobile health (mHealth) is an interesting and ever-expanding resource for disseminating health information and services. In 2016, the World Health Organization mHealth Technical Evidence Review Group developed a checklist for reporting health interventions that utilize cell phones [8]. The CHARISMA study showed that mobile technologies can support medical care services and prevention in a resource-efficient manner [9]. The coronavirus pandemic accelerated mHealth interventions, as digital media became an integral component of the public health care system and acceptance of digital health care services by the public increased [10]. We hypothesized that a comprehensive information on effective and individualized prevention strategies in a single, easily accessible, Web-based tool could improve informed decision-making by the eligible population and potentially encourage the adoption of effective prevention strategies. A centrally administered web portal enables easy access to health care, transfer of knowledge, and the rapid adaptation of health information [11]. Another aspect is the fact that a web application could help to raise awareness for screening and prevention also in harder-to-reach populations [12]. Web application users should be able to assess their individual risk for three common types of cancer, breast, prostate, and colorectal cancer based on recommendations from the current national, German S3 guidelines and receive individualized suggestions. The web application aims at individuals who are eligible for, but have not yet taken any preventive measures or individuals with a higher cancer risk, such as those with a family history of cancer. The web application provides customized information about the individual risk (as assessed by life style factors and family history) as well as decision-relevant information on screening and early detection examinations, including behavior-related preventive measures, their benefits, as well as potential risks associated with the examinations themselves.

### Objectives

Here, we describe the pilot evaluation of this web-based, interactive tool for guideline-based, risk-adapted information on preventive measures for colorectal, breast, and prostate cancer by the first 101 anonymous users. The major goals were to assess (1) the usability and user friendliness of the web application on cross-entity cancer prevention and (2) whether the information received through the web application would motivate users to seek more information on preventive measures.

## Methods

### Development of the Web Application

The web application is structured as an interactive questionnaire for the prevention related content. Rule-based artificial intelligence was used to dynamically adjust subsequent questions based on the participants’ real-time responses. This approach aims to reduce the time required for users by narrowing the questionnaire to a set of questions that are personally relevant to the topic. This questionnaire (see Multimedia Appendix 1) and its underlying rule set were developed by a multidisciplinary team based on the recommendations regarding screening and early detection measures for colorectal, breast, and prostate cancer in the respective German S3 guidelines (version 2.1 from 2019 for colorectal cancer [13], version 4.4 from 2021 for breast cancer [14], and version 6.2 from 2021 for prostate cancer [15]). The respective rules are provided in Multimedia Appendix 2. The multidisciplinary team comprised members from the departments of General Medicine, Urology, Gynecology, and Gastroenterology at Ulm University Hospital, as well as experts from the Institute of Medical Systems Biology at Ulm University’s Medical Faculty. The application was designed with a focus on straightforward usability, and its responsive design ensures high compatibility across various devices and screen sizes. The evaluation tool was also developed by the multidisciplinary team. It comprised 10 more “technical” questions that were taken from the publicly available system usability scale (SUS) questionnaire [16] that was translated into German. The SUS was supplemented with 10 specific questions regarding the user experience with the web application that was also developed by the multidisciplinary team. The questions were answered on a five-point Likert scale ranging from “strongly disagree” to “strongly agree.” Some of the evaluation questions were phrased positively, while others were phrased negatively, and for this reason, they were evaluated individually.

**Figure 1.**
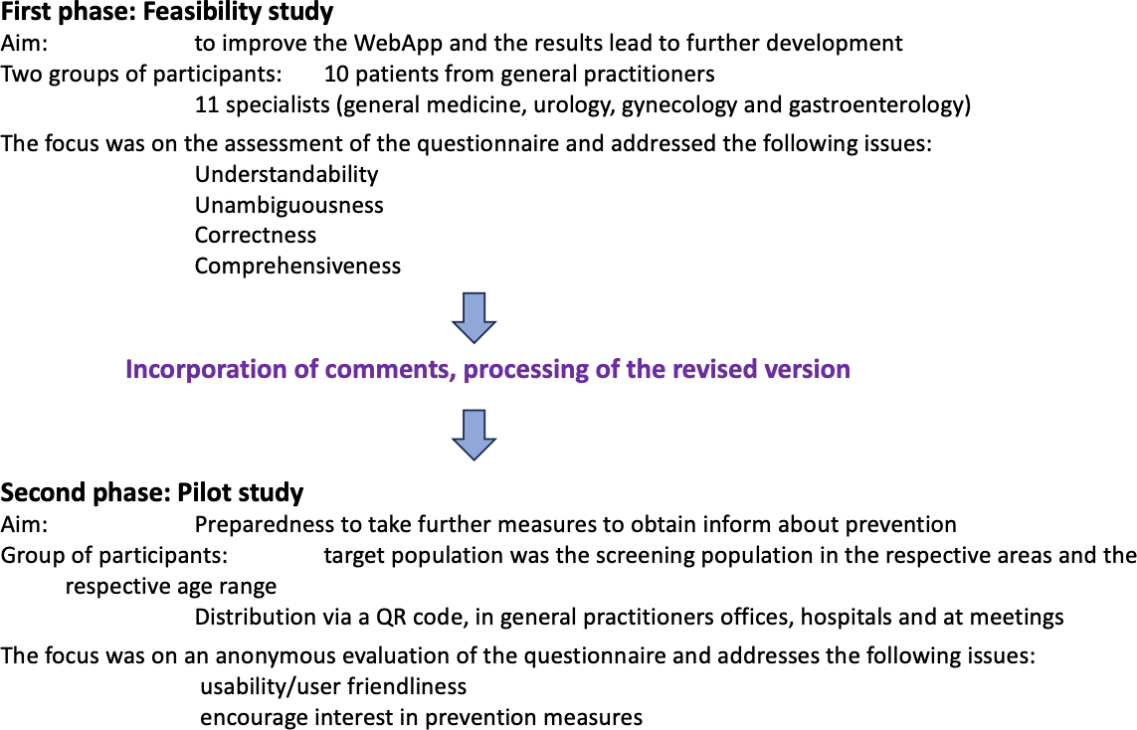
Schema of the feasibility and pilot study.

The web application as well as the evaluation questionnaire were first evaluated by two focus groups in a feasibility study. The groups consisted of 10 patients, 50‐70 years of age with no previous history of cancer from participating general practitioners and 11 specialists from general medicine, urology, gynecology and gastroenterology, respectively. None of the participating doctors had been involved in the app’s development. These two groups focused on understandability, unambiguousness, correctness and comprehensiveness of the content as well as the evaluation questions. The individual feedback from the two groups was discussed by the entire Prevent-Take-Up team, and both content-and evaluation-related suggestions for improvement were incorporated into the web application to enhance usability. The results of this evaluation are published elsewhere [17]. The web application was subsequently revised and the revised version was made publicly available to a screening population for phase 2 (Figure 1) which was the topic of this manuscript.

### Recruitment of the Study Population

For recruitment of participants posters and flyers were distributed in general practitioners’ offices and hospitals in the State of Baden Württemberg at Ulm University Hospital in southern Germany. In addition, these brochures were also distributed at meetings (ie, Annual conference of the Southwest German Society for Gastroenterology, German Cancer Congress in Berlin) containing a QR code that led to the entire web application questionnaire, comprising both content- and evaluation-created questions. While we were aiming for a screening population which was clearly stated on the promotion material provided, use of the web application was entirely anonymous and we could, therefore, not assess whether this criterion was met. Participants first answered the content-related questions and received a personalized recommendation. Subsequently, the web application asked the participants to provide a voluntary, anonymous feedback on the usability and functionality of the application via this tool. These participants did not receive any compensation for their participation.

### Measures

The web application considers sex, age, individual risk and lifestyle factors, including physical activity, alcohol, and nicotine consumption (Table 1), as well as comorbidities and past medical check-ups. Queried previous investigations included colonoscopy, a fecal occult blood test (FOBT), mammography, prostate magnetic resonance imaging (MRI) test, digital prostate examination, or a prostate-specific antigen (PSA) test up to this point in time. Guideline-recommended time intervals for repeating specific screening tests such as mammography or colonoscopy were also considered. A family history of cancer was queried for first-degree relatives (ie, parents, siblings, children). Recommendations for colorectal cancer screening also took into account polyps removed during a previous colonoscopy or the results of a FOBT.

**Table 1.** Characteristics of the study population (N=101).

Characteristics	Participants (N=101)
Sex^a^, n (%)	
Male	38 (38)
Female	62 (62)
Age range (years), n (%)	
50‐70	93 (92)
<50	8 (8)
BMI, n (%)^b^	
<18.5	0 (0)
18.5 to <25	29 (29)
25 to <30	44 (43)
≥30	26 (26)
METs^c^, n (%)	
>150/ week	77 (76)
Alcohol consumption, n (%)^d^	
No	59 (58)
Yes	41 (41)
1‐5 days/week	28 (28)
6‐7 days/week	6 (6)
≤12 g per day	2 (2)
>12 g per day	2 (2)
Nicotine consumption, n (%)	
No	73 (72)
Yes	28 (28)
Red meat consumption, n (%)	
No	59 (58)
Yes	42 (42)
Diabetes mellitus, n (%)	
No	86 (85)
Yes	8 (8)
Don´t know	6 (6)

a One person did not specify the sex; a total of 100 out of 101 people provided.

b BMI data for 2 persons could not be provided; therefore data for a total of 99/101 is presented.

c METs: metabolic equivalent of tasks.

d One person did not report alcohol consumption.

The metabolic equivalent of a task (MET) was used as a measure of physical activity [18]. Participants assessed their own physical activity and classified themselves as either “physically active with more than 150 MET minutes per week” or “physically inactive with less than 150 MET minutes per week.” Participants were also asked about nicotine, red meat and alcohol consumption, the frequency of alcohol intake (1‐5 d or 6‐7 d per wk) and the amount of alcohol consumed (≤12 g/d for women and ≤24 g/d for men) [19]. Diabetes mellitus was also queried (Yes/No/Don’t know). All answers were taken into consideration for the personalized recommendations. Based on all information provided, the web application recommended to consult a GP regarding specific preventive procedures, if at least one of the recommended preventive measures was outside the recommended timeframe, had not been completed to date, or if alerting symptoms were reported.

Participants were also asked to group themselves according to sex and age (<30, 30‐50, and 50‐70 y). BMI was calculated from the height and weight data and categorized according to the WHO classification as underweight (BMI <18.5), normal weight (BMI 18.5 - <25), preobese (BMI 25 - <30), and obese (BMI≥30) [2]. The medical questionnaire comprises 21 questions, with the option to skip questions, and is provided as an additional file (Multimedia Appendix 1).

Having completed the medical questionnaire, participants were invited to take part in a survey in which they were asked to rate the functionality and user-friendliness of the web application. A questionnaire was developed for this in which the questions from the publicly available SUS questionnaire [16] were supplemented with thematic questions about the web application and agreed upon within the project team. Participants were also asked whether they found the information provided by the website application helpful, if they would use it, and if they would follow the recommendation to seek further medical advice, eg, from their GP.

Results are presented purely descriptively and separately for male and female participants. Participation in screening programs for colon cancer differs between males and females [20]. Due to the fact that breast cancer occurs very rarely in men and prostate cancer only in men, we decided to evaluate the questionnaires by sex to identify possible sex specific differences that could be potentially addressed in further developments of a web application. The only non-descriptive statistic used was to elucidate whether the recommendation to consult a GP was driven by the website application rather than being the result of a random selection. For this purpose, we employed a binomial test to assess this deviation from a random recommendation (statistical software R version 4.4.2).

The trial was registered in the German Clinical Trials Register (DRKS00029631) and the software implementing the Prevent-Take-Up web application is freely available [21].

### Ethical Considerations

The project was approved by the Ethics Committee of Ulm University (ethics committee No. 122122, 2022). All data were entered voluntarily and anonymously by participants who scanned the QR code and completed the web-based questionnaire. Therefore, a signed consent form was not obtained but completion of the Prevent web application questionnaire was considered as consent to participate. The study data are therefore anonymous, and the participants are unknown and not traceable. These participants did not receive any compensation for their participation.

## Results

### Baseline Characteristics Analysis

A total of 101 participants tested the web application; 62% (n=62) were female and 38% (n=38) were male; one participant did not specify sex. A total of 91% (n=92) of the participants were older than 50 years and had the age of the screening population; 1/93 participants (1%) did not specify sex, but their age was >50 years. Since participation was entirely anonymous, we would not have been able to verify inclusion or exclusion criteria. However, it was advantageous that individuals younger than the target population participated in the evaluation (8/101, 8%; 4 female and 4 male) as future screening programs may also be available for younger individuals with a family history (eg, of colorectal cancer). Regarding BMI, the majority of the study population was in the preobese range, with a median BMI of 26 (IQR 22.6‐29.3), and 28.3 (IQR 26.06‐31.25) for females and males, respectively (Table 1). Overall, 98 of 101 participants (97%) provided a complete dataset, while the response rate for the remaining 3 participants (3%) exceeded 90%.

### Assessing Risk Factors

The content questionnaire included several questions on lifestyle factors. Physical activity was assessed asking for the duration of METs. 30/38 (79%) of male and 47/62 (76%) of female participants reported being physically active for more than 150 MET minutes per week. Alcohol consumption was reported by 20/61 (32%) females and 20/38 (53%) males. Specifically, 15/20 (75%) females and 20/38 (65%) males in the “consumer group” indicated consuming alcohol on 1‐5 days per week, 1/20 (5%) females and 5/20 (25%) males on 6‐7 days per week. More specific amounts of alcohol intake were reported only by a few participants: 2/20 (10%) females reported a daily intake of less than 12 g, 2/20 (10%) males of up to 24 g of alcohol. Current nicotine use was reported by 19/62 (31%) female participants and 9/38 (24%) male participants. Additionally, 25/62 (40%) females and 16/38 (42%) males consumed red meat regularly. Diabetes mellitus was reported by 2/61 (3%) females and 6/38 (16%) males. 57/61 (92%) female and 29/38 (76%) male participants denied having diabetes mellitus, 2/61 (3%) females and 3/38 (8%) males were unsure about this issue, and 1/61 (2%) female participant did not respond to this question (Table 1).

### Evaluation of the Content Questionnaire and the Web Application by the Participants

**Figure 2.**
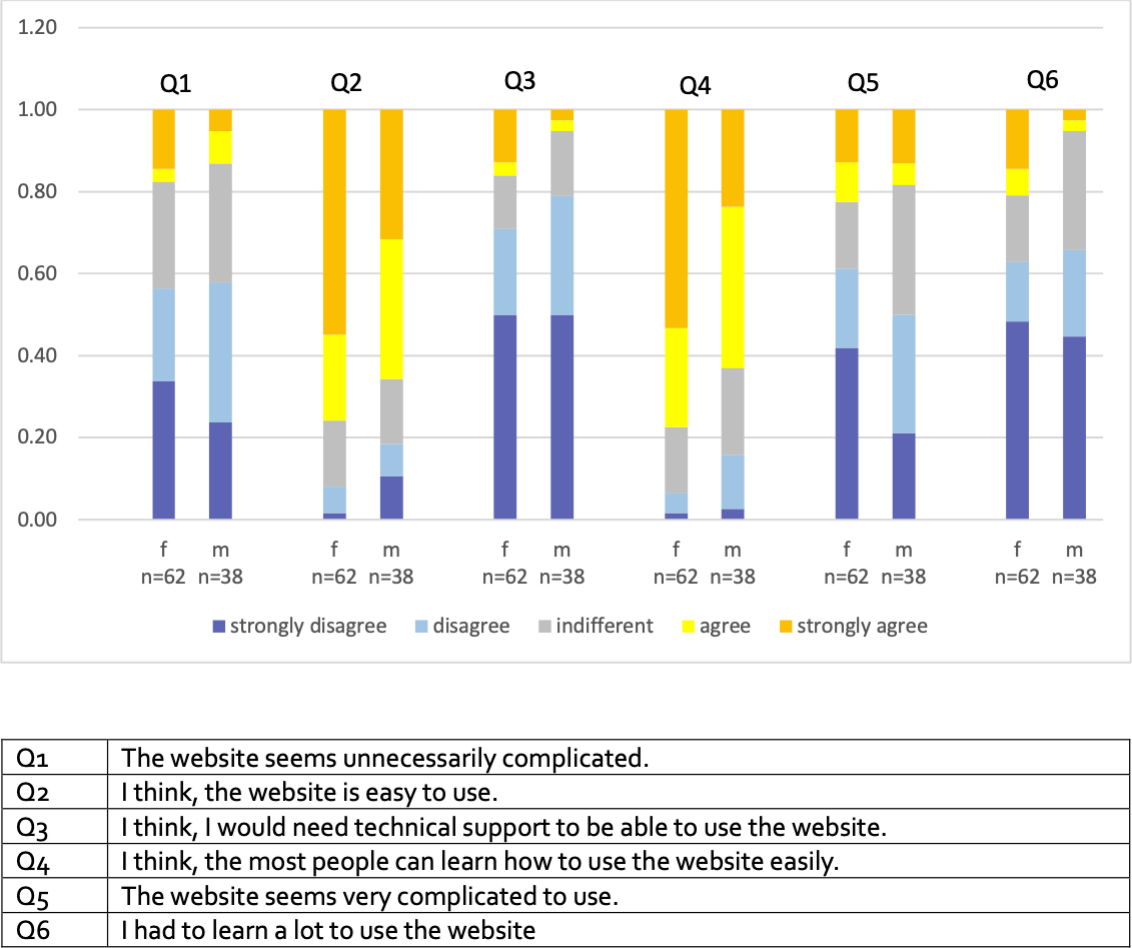
Technical usability of the prevent-take-up app. f and m indicate females and males, respectively.

The main focus of this manuscript is the evaluation of the web application and its content questionnaire by the participants. Usability was rated as “easy” (agree or strongly agree) by 47/62 (76%) females and 25/38 (65%) males. 48/62 (77%) of female and 24/38 (63%) male participants agreed or strongly agreed that most people can quickly learn to use the application. 44/62 (71%) females and 30/38 (79%) males stated that technical support was not necessary for using the application (Figure 2).

**Figure 3.**
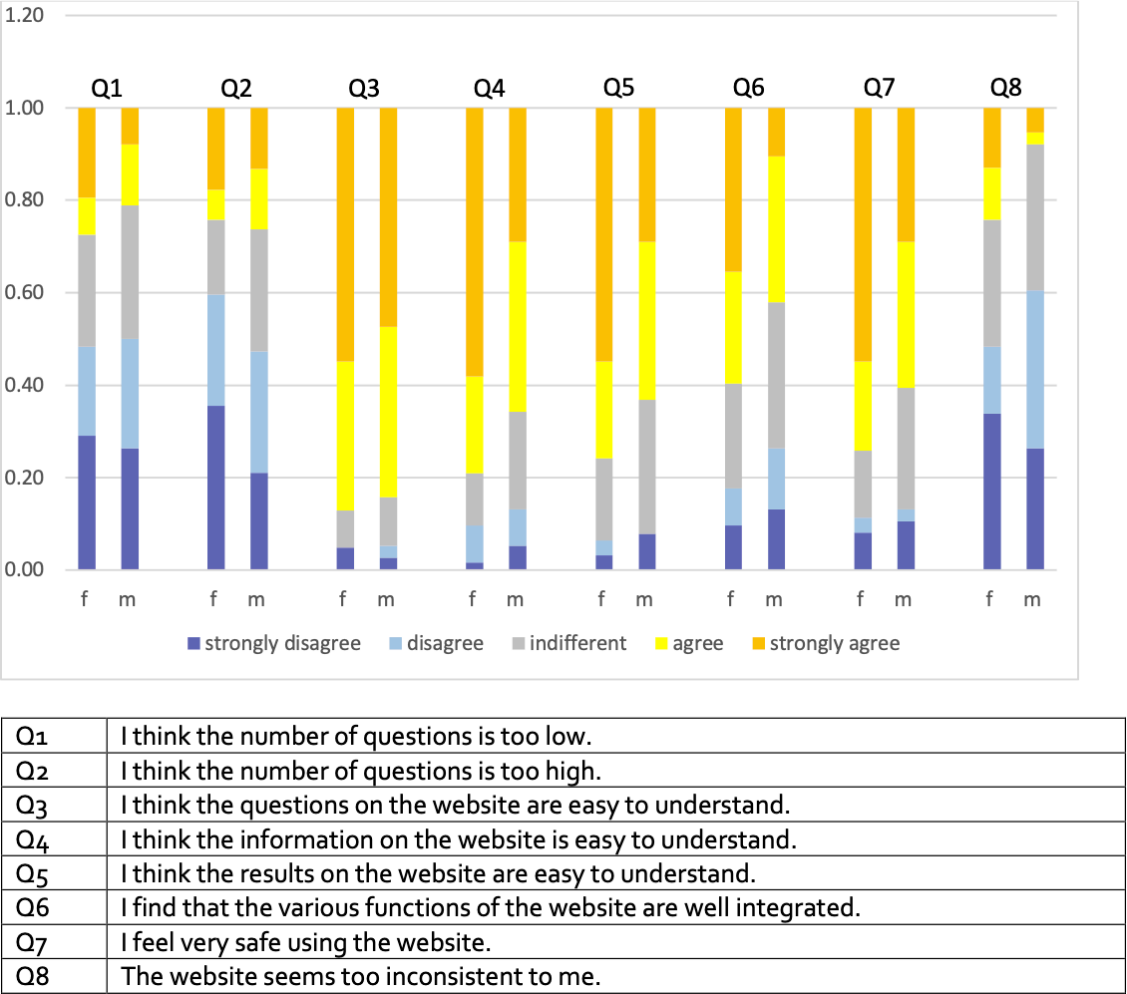
Assessment of the questionnaire and functionality. f and m indicate females and males, respectively.

The majority of female and male participants agreed or strongly agreed that the questions posed (female: 54/62, 87%; male: 32/38, 83%) and the recommendations provided (female: 47/62, 76%; male: 24/38, (63%). Participants had access to additional information on screening and preventive measures within the web application. 49/62 (79%) females and 25/38 (66%) males agreed or strongly agreed that the informative texts in the web application were clear (Figure 3).

37/62 (59%) of female and 16/38 (44%) of male participants agreed or strongly agreed that the app’s various functions were well integrated. 30/62 (49%) of female and 23/38 (59%) of male users totally disagreed or disagreed that the web application appeared too inconsistent while 46/62 (74%) females and 23/38 (60%) males agreed or totally agreed that they felt safe using the web application (Figure 3).

**Figure 4.**
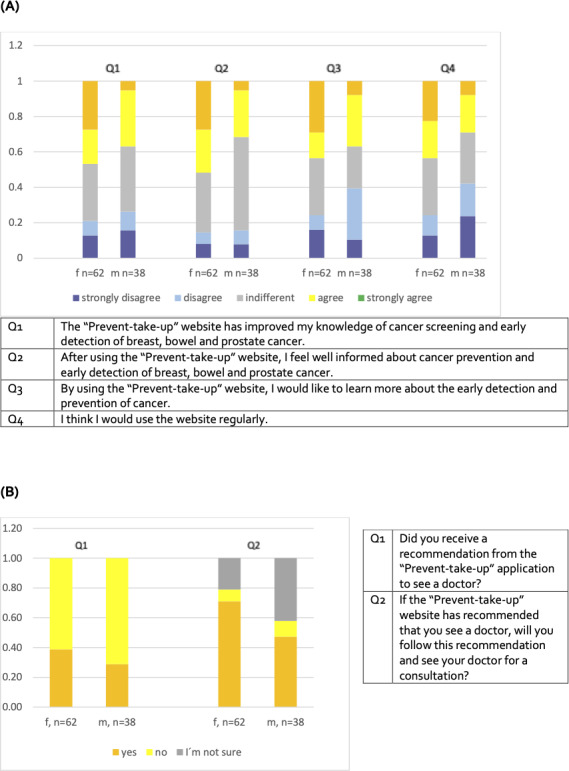
Improvement of the understanding of cancer screening and taking action after screening recommendation. (A) Level of knowledge; (B) Screening recommendation. f and m indicate females and males, respectively.

29/62 (47%) of female and 14/38 (37%) of male participants agreed or strongly agreed that the web application improved their understanding of cancer screening and early detection in the respective areas. 32/62 (52%) female and 12/38 (32%) male participants agreed or strongly agreed that they felt more informed about prevention and early detection of breast, bowel, and prostate cancer after using the web application. 27/62 (44%) female and 14/38 (37%) male participants agreed or strongly agreed that they used the web application to learn more about early detection and prevention of cancer. Furthermore, 27/62 (44%) female and 11/38 (29%) male participants agreed or strongly agreed that they would use the app regularly, most likely because changes in individual lifestyle factors over time may lead to different risk assessments (Figure 4A).

24/62 (39%) females, 11/38 (29%) males, and one individual without an indication of sex received a recommendation to consult their GP regarding further information on preventive check-ups based on their “prevention history” as well as individual lifestyle and risk factors. Binomial testing showed that these recommendations were indeed not given by chance, but driven by the web application algorithm (*P*=.005, two-tailed, binomial test). Furthermore, 44/62 (71%) female and 18/38 (47%) male participants indicated they would follow the application’s recommendation to see their GP for more information on preventive measures. Others stated they would not follow this recommendation whereas 13/62 (21%) females and 16/38 (42%) males expressed uncertainty about following the recommendation (Figure 4B).

Of note, in many items of the questionnaire, female participants compared to male participants provided a numerically more positive assessment of the web application and stated greater willingness to follow the recommendations given.

## Discussion

### Main Findings

This web application was developed to provide low-threshold, cross-entity and individual risk-adjusted information on cancer prevention. It is an evidence-based tool, developed by experts in the respective fields in Germany. The web application does not directly give a medical advice but makes individualized recommendations to seek further advice from their GPs or respective experts. The web application was highly rated by the majority of participants. A majority—at least 71% of female and 47% of male participants—stated that they would take advantage of an information session with their GP after receiving a respective recommendation by the web application.

### Comparison With Prior Work

There is an increasing number of apps in the area of cancer prevention, including customized text messages or fotonovela programs that encourage screening uptake, eg, for colorectal or breast cancer [22-24]. The majority of these apps focuses on prevention of a particular tumor or give advice on how to perform a specific preventive test such as an FOBT [25]. Very few apps take into account individual risk factors such as exercise, smoking, obesity, or alcohol consumption when making a recommendation for a preventive test. Lifestyle changes and behavioral prevention can reduce cancer incidence [26]. A meta-analysis showed a 10‐20 percent reduction in the risk of developing various cancers in physically active individuals [27]. Reporting risk factors such as consumption of alcohol, red/processed meat or low physical activity is more likely to be done anonymously online, rather than in a face to face interview [28]. Thus, a web application like Prevent-Take-up could encourage individuals to take proactive steps toward primary prevention by highlighting the benefits of specific lifestyle changes, such as increasing physical activity. It is well known that knowledge of cancer prevention, screening tests, and individual risk factors can motivate the eligible population to take part in screening [29].

This was the goal of our web application. We did not intend to produce a medical device or a diagnostic tool according to the In-Vitro Diagnostic Regulation. Therefore, the strongest recommendation by the app was to seek further advice from a trusted expert, which was GP for the majority of the population. Structured integration of GPs into an overarching prevention program is an important step to improve informed decision making on participation in cancer screening and preventive measures.

It remains a challenge to disseminate information on prevention to the less informed part of the population. It is remarkable that particularly younger and potentially digitally more savvy people (<50) felt well-informed simply by using the web application, but the numbers are low. Social media may not be the only solution since, in a survey from Canada, no correlation was found between low knowledge of guideline-based screening and participation in screening programs, although participants were willing to link their information to their medical records [30-33]. Therefore, we included GPs as trusted experts in the entire process.

The reported nicotine consumption by 31% of female and 24% of male participants is higher than that reported in the 2021 microcensus (15.7% of women and 22.3% of men) [1] that included all age groups. Individuals using the web application may be less health-conscious. Alternatively, since the survey was mainly advertised in health care facilities, participants of the survey may have been there for treatment of medical conditions potentially also due to nicotine consumption [34].

Generating a web application rather than an app in an app store has significant benefits for an academic group: Updates can easily be made in case of small changes to screening programs; it offers greater flexibility to developers since it is not bound by the regulations imposed by commercial app stores and it is far cheaper to maintain [35].

### Strengths and Limitations

A strength of the study is the approach to inform about preventive measures in a comprehensive, cross-entity manner and at the same time take individual risk factors like family history, comorbidities, and lifestyle factors into account to recommend a risk-adapted consultation by the GP. This recommendation could also save the family doctors’ time since critical individual topics are already identified and can be directly addressed. Another strength is the involvement of GPs from the beginning in both the design and the realization of the study. GPs are persons of trust and are an important, yet underused, partner in the whole spectrum of cancer prevention. GPs can make a major contribution to informed decision-making.

Our study has also several limitations. First, the participant group was small with only n=101 individuals taking the survey. Second, the individuals who participated in the surveys were probably mainly recruited from general practices where the study was advertised. There might be a bias towards participants with previous conditions such as obesity, and participants may not reflect the average population. Third, participation was anonymous making it impossible to determine whether the participants were representative of the screening population, whether the answers given were true, and disabling any follow-up. The questionnaire could hence only evaluate intention, which does not equate to behavior of taking up a preventive measure.

Finally, 47% of female and 37% of male participants stated that their knowledge of preventive and early detection measures had increased due to the web application. This rate is below 50% and clearly demands further improvements; however, it could also reflect a selection bias due to an already knowledgeable portion of the population participating in the survey.

### Conclusions

Prevent-Take-up is a simple web application designed to improve informed decision-making on comprehensive screening for frequent tumor entities using guideline-based recommendations. The web application was highly rated and well accepted by the target population. Thus, Prevent-Take-Up follows a sensible approach.

### Future Directions

Translating the content of the web application in various languages could help bridge language barriers and facilitate better communication with GPs and specialists. The web application could also be expanded to include a feedback function that allows participants to report whether the recommendations were followed in an anonymous fashion and the respective outcomes. Finally, the study could be extended to incorporate a larger group and also potentially younger people with a higher risk of cancer due to individual risk factors. The challenge is to reach as many individuals possible who are eligible for different reasons such as age, family or individual risk. In particular, the implementation of strategies to ensure equitable access for different population groups is still challenging [36]. The active involvement of potential users in the development of this web application was crucial and will contribute to the continued improvement of Prevent-Take-Up.

## Supplementary material

10.2196/76393 Multimedia Appendix 1 Questions in the questionnaire regarding prevention.

10.2196/76393 Multimedia Appendix 2 Rule table for the app.

## Abbreviations

FIT: fecal immunochemical test
FOBT: fecal occult blood test
GP: general practitioner
MET: metabolic equivalent of task
mHealth: mobile health
MRI: magnetic resonance imaging
PSA: prostate-specific antigen
S3 guideline: guidelines with recommendations based on evidence or expert consensus
SUS: system usability scale
