# Supplementary material for: Integrated, Cross-Entity Information on Preventive Measures for Bowel, Breast, and Prostate Cancer: Evaluation Study of the Web Application “Prevent-Take-Up”
Source: JMIR Cancer. 2025 Nov 19;11:e76393. doi: 10.2196/76393 (PMC12630371; doi:10.2196/76393)
Supplement: Multimedia Appendix 1 [file cancer-v11-e76393-s001.docx]

**Questionnaire**

**Questionnaire regarding prevention**

**General part:**

- What gender are you?
- How old are you?
- What is your body weight (in kg)?
- What is your height (in cm)?
- Open feedback

Questions about lifestyle/pre-existing conditions:

- Have you ever had cancer?
- Do you eat red meat several times a week?
- Please rate your energy usage per week and then select the answer that suits you best?
- Do you smoke?
- Do you drink wine/beer/sparkling wine/spirits? If so:

Female: How often do you drink up to 1 glass of wine (125 ml) or beer (300 ml) or 2 glasses of spirits (40 ml) per day?

Male: How often do you drink up to 2 glasses of wine (250 ml) or 2 glasses of beer (600 ml) or 4 glasses of spirits (80 ml) per day?

- Do you have diabetes mellitus type II?

Screening examinations:

- Which preventive check-ups have you already taken part in?

Health check-up, skin cancer screening, colonoscopy, palpation of the female breast, ultrasound examination of the female reproductive organs, ultrasound examination of the prostate, PSA value?

When were these?

**Family history**

- Are there or have there been several cases of cancer in your family?

- Do you have a known genetic predisposition to cancer?

Mother:

- Does your mother have a family history of cancer?
- What cancers have occurred in your mother's family?
- At what age did your mother or your mother's relatives develop cancer for the first time?

Father:

- Did cancer occur in your father's family?
- Which cancers occurred in your father's family?
- At what age did your father or your father's relatives develop cancer for the first time?

Siblings:

- Did your siblings develop cancer?
- Which cancers have occurred in your siblings?
- At what age did one or more of your own children develop cancer?

**Specific questions on each of the tumor entities:**

Screening for colorectal cancer:

- Have you ever had a test for “blood in the stool” (Hämoccult®, iFOPT)?

If yes: which test? Positive or negative? If positive, was a colonoscopy also performed? If yes, when exactly was the colonoscopy

- Have you had a colonoscopy in the last 10 years?

If yes: Were precancerous lesions (e.g. polyps, adenomas) removed during the colonoscopy?

Was colon cancer detected during the colonoscopy?

Have you or one of your relatives had more than 1 polyp or adenoma removed during the same examination?

Breast cancer screening:

- Have you felt lumps in your breast?
- Have there been cases of breast cancer in your family?
- Who in your family has had breast cancer and at what age?
- Have there been any cases of ovarian cancer in your family?
- Have you yourself been diagnosed with breast cancer? If yes:

Were you diagnosed with breast cancer more than 5 years ago?

- When was your last check-up at your gynecologist?
- When was your last mammography?

Screening for prostate cancer:

- How do you feel about early detection of prostate cancer?
- When did you have your screening test?
- How high was the PSA value?

**Questionnaire regarding assessment of the usability/user friendliness**

Technical usability (Figure 1):

Q1: The website seems unnecessarily complicated.

Q2: I think, the website is easy to use.

Q3: I think, I would need technical support to be able to use the website.

Q4: I think, the most people can learn how to use the website easily.

Q5: The website seems very complicated to use.

Q6: I had to learn a lot to use the website.

Functionality (Figure 2):

Q1: I think the number of questions is too low.

Q2: I think the number of questions is too high.

Q3: I think the questions on the website are easy to understand.

Q4: I think the information on the website is easy to understand.

Q5: I think the results on the website are easy to understand.

Q6: I find that the various functions of the website are well integrated.

Q7: I feel very safe using the website.

Q8: The website seems too inconsistent to me.

Knowledge (Figure 3A):

Q1: The “Prevent-take-up” website has improved my knowledge of cancer screening and early detection of breast, bowel and prostate cancer.

Q2: After using the “Prevent-take-up” website, I feel well informed about cancer prevention and early detection of breast, bowel and prostate cancer.

Q3: By using the “Prevent-take-up” website, I would like to learn more about the early detection and prevention of cancer.

Q4: I think I would use the website regularly.

Recommendation (Figure 3B):

Q1: Did you receive a recommendation from the “Prevent-take-up” application to see a doctor?

Q2: If the “Prevent-take-up” website has recommended that you see a doctor, will you follow this recommendation and see your doctor for a consultation?
